# Does preferred technique influence how kinematics change during a run to exhaustion?—A cluster based approach

**DOI:** 10.7717/peerj.20673

**Published:** 2026-02-11

**Authors:** Adrian R. Rivadulla, Zak Sheehy, Xi Chen, Dario Cazzola, Grant Trewartha, Ezio Preatoni

**Affiliations:** 1 Department for Health, University of Bath, Bath, United Kingdom; 2 Department of Computer Science, University of Bath, Bath, United Kingdom; 3 School of Health & Life Sciences, University of Teesside, Middlesbrough, United Kingdom

**Keywords:** Running performance, Fatigue, Athletics, Unsupervised learning, Functional groups

## Abstract

Fatigue-related changes in running technique may depend on a runner’s preferred style. Understanding these changes can inform targeted training to enhance performance. In previous work, we identified two technique-based clusters of runners: the “neutral pelvis” and the “tilted pelvis” clusters. This follow-up study examined whether fatigue induces cluster-specific technique adaptations. Sixty runners (neutral pelvis, *n* = 32; tilted pelvis, *n* = 28) completed a treadmill run to exhaustion at 5% above their individual lactate threshold speed. Stride frequency, duty factor, trunk and lower limb kinematics were compared between clusters at the start, middle, and end of the run using a 2-way repeated measures analysis of variance (ANOVA). All runners reached exhaustion in ∼20 minutes, covering ∼5 km. Runners from the tilted pelvis cluster consistently showed greater trunk-to-pelvis extension, more pelvic anterior tilt and greater hip flexion, and a smaller duty factor compared with the neutral pelvis cluster throughout the run. Fatigue-related adaptations were similar across clusters: reduced stride frequency, increased duty factor, greater trunk flexion during stance, increased plantar flexion, and higher coordination variability (trunk-to-pelvis–hip, hip–knee, knee–ankle) during swing. Although fatigue affected both groups similarly, the underlying technique differences suggest these adaptations may have distinct mechanical or performance consequences. Understanding such cluster-specific responses can help coaches tailor training and fatigue management strategies to individual running styles.

## Introduction

In endurance sports like running, athletes competing to achieve their best performance encounter a common foe: fatigue. Fatigue is a multifaceted phenomenon with physiological (McKenna & Hargreaves, 2008), biomechanical (Apte et al., 2021; Zandbergen et al., 2023) and cognitive (Hampson et al., 2001) implications, which emerges as a critical determinant of running performance and injury susceptibility (Winter, Gordon & Watt, 2017). As athletes engage in prolonged or intense bouts of running, the acute manifestation of fatigue is an inability to sustain their pace, leading to slowing down in free running or to the termination of a constant speed running trial (Chan-Roper et al., 2012; Strohrmann et al., 2012). Over the longer term, balance between fatigue and recovery stimuli during training can promote positive adaptations leading to running performance gains (Kellmann et al., 2018). On the contrary, inappropriate fatigue management has been suggested as a contributor to the occurrence of running injuries, especially overuse injuries related to repetitive loading of the musculoskeletal tissue (Winter, Gordon & Watt, 2017). Understanding the intricate relationship between fatigue, running performance and injury is paramount for athletes, coaches, and healthcare professionals seeking to optimise training strategies, mitigate injury risk, and enhance overall athletic outcomes.

As a fundamental aspect of human physiology, fatigue encompasses the complex interplay of metabolic, neuromuscular, and psychological factors that ultimately dictate the capacity of an athlete to sustain exercise (Enoka & Duchateau, 2008; Enoka & Duchateau, 2016; Enoka & Stuart, 1992; McKenna & Hargreaves, 2008). Fatigue has been described as “a disabling symptom in which physical and cognitive function is limited by interaction between performance fatigability and perceived fatigability” (Enoka & Duchateau, 2016). Fatigue symptoms are associated with alterations to running biomechanics which have received considerable attention in the literature. Four recent reviews (Apte et al., 2021; Kim, Mirjalili & Fernandez, 2018; Winter, Gordon & Watt, 2017; Zandbergen et al., 2023) summarised fatigue-induced changes in kinetics (*e.g.*, lower peak vertical ground reaction force), kinematics (*e.g.*, greater trunk flexion, increased hip and knee range of motion, higher knee flexion and lower ankle plantarflexion angles at initial contact), spatiotemporal parameters (*e.g.*, decreased stride frequency, longer contact times) and muscle activity (*e.g.*, decreased integrated EMG of the hamstring and the calf). Nevertheless, the direction of those changes is subject to some controversy. For instance, whilst reduced stride frequency and increased contact times seemed well-established changes to technique in the literature (Apte et al., 2021; Zandbergen et al., 2023), Kim, Mirjalili & Fernandez (2018) identified conflicting findings in their review. Fatigue can also affect joint and segment coordination and variability (Mo & Chow, 2019; Möhler et al., 2020; Tazji et al., 2023). An optimal level of coordination variability is thought to be associated with a healthy state. On the other hand, too little variability can lead to overloading of the same structures and too much variability may reflect instability or lack of control of the system (Hamill et al., 2006). Skill level plays a key role in the changes to technique associated with fatigue, with recreational runners usually displaying larger adjustments to their technique compared with experienced runners (Maas et al., 2018; Strohrmann et al., 2012; Zandbergen et al., 2023). Methodological differences and limited sample sizes in previous studies may have also contributed to the inconsistencies in the reported biomechanical changes associated with fatigue (Apte et al., 2021; Kim, Mirjalili & Fernandez, 2018; Winter, Gordon & Watt, 2017; Zandbergen et al., 2023). For instance, most studies investigating fatigue and running technique have used exhaustion protocols where the rate of perceived exertion is measured (Apte et al., 2021). However, the magnitude and direction of running technique changes associated with fatigue can be affected by the intensity of the run (Apte et al., 2021), making it critical to control for this factor in experiments when possible.

Runners are also thought to develop a preferred technique through years of training (Nigg et al., 2017; Williams & Cavanagh, 1987), which may result in different athletes exhibiting different adaptations of their technique with fatigue (Apte et al., 2021). In recent work (Rivadulla et al., 2024) we have used unsupervised learning to identify two clusters of runners, the “neutral pelvis” and the “tilted pelvis” clusters, who showed differences in their preferred technique: the tilted pelvis cluster exhibited greater trunk-to-pelvis extension, more anterior pelvic tilt and greater hip flexion throughout the stride as well as smaller duty factor compared with the neutral pelvis cluster. Despite the kinematic differences, these two groups did not show differences in their running economy, demographics, anthropometrics, physiological capacity, training load and performances. Understanding whether runners from different clusters exhibit different changes in their technique due to fatigue could reveal important implications for the development of training and injury prevention/rehabilitation interventions.

Building on our previous research where we identified the neutral and tilted pelvis clusters of runners (Rivadulla et al., 2024), the aim of the present study was to compare the changes in technique of the runners from these clusters during a run to exhaustion. It was hypothesised that runners from each cluster would exhibit different technique modifications throughout the run. As secondary objectives and in support of the main aim of the study, we investigated the differences in running technique between clusters and technique adjustments to exhaustion across all runners.

## Materials & Methods

Sixty members of the two clusters (neutral pelvis, *n* = 32; tilted pelvis, *n* = 28, Table 1) identified in our previous study came back for a second test ∼7 days after the incremental test described in Rivadulla et al. (2024). All participants were between 18 and 50 years old, had not experienced any injuries in the six months leading up to the study, and reported recent 10K race times under 57 min and 20 s for women and under 50 min for men for matched age-grading (USATF, 2020). Participants were recruited *via* social media, flyers on the University of Bath campus and local running clubs. Every participant provided written informed consent before performing the test, and the study received ethical clearance from the University of Bath’s Research Ethics Approval Committee for Health (REACH, EP 18/19 064).

**Table 1:** Runners’ descriptive statistics.

**Variable**	**All**		**Neutral pelvis**	**Tilted pelvis**	**Cluster comparison**
	**F**	**M**			
Sample (n)	60		32	28	
**Demographics**			
Sex (n, %)	25 (41.7%)	35 (58.3%)	16 F (50%)	9 F (32.1%)	*X*^2^ = 1.29, *p* = 0.26
Age (years, $\overline{x}(s)$)	33 (10)	35 (10)	35 (10)	33 (10)	U = 504.5, *p* = 0.41
**Anthropometrics**				
Height (m, $\overline{x}(s)$)	1.66 (0.04)	1.79 (0.06)	1.72 (0.08)	1.75 (0.09)	t = −1.32, *p* = 0.19
Body mass (kg, $\overline{x}(s)$)	56.5 (5.8)	72.2 (6.7)	63.6 (9.5)	68.1 (10.2)	U = 339, *p* = 0.11
Trunk length (m, $\overline{x}(s)$)	0.72 (0.04)	0.81 (0.04)	0.77 (0.06)	0.78 (0.06)	t = −1.09, *p* = 0.28
Leg length (m,$\overline{x}(s)$)	0.93 (0.04)	0.98 (0.05)	0.95 (0.05)	0.96 (0.05)	t = −0.89, *p* = 0.38
Pelvis width (m, $\overline{x}(s)$)	0.16 (0.01)	0.17 (0.01)	0.17 (0.01)	0.17 (0.01)	t = 0.26, *p* = 0.80
Thigh length (m, $\overline{x}(s)$)	0.42 (0.02)	0.44 (0.02)	0.43 (0.02)	0.43 (0.03)	t = −0.41, *p* = 0.68
Shank length (m, $\overline{x}(s)$)	0.38 (0.02)	0.42 (0.02)	0.40 (0.03)	0.41 (0.03)	t = −0.42, *p* = 0.68
Foot length (m, $\overline{x}(s)$)	0.26 (0.01)	0.28 (0.01)	0.27 (0.02)	0.28 (0.02)	t = −0.98, *p* = 0.33
**Physiology**					
$\dot {V}{O}_{2}peak$ (ml/min/kg, $\overline{x}(s)$)	52 (5.4)	51.7 (6.5)	51.5 (6.1)	52.1 (5.9)	t = −0.39, *p* = 0.69
LT (km/h, $\overline{x}(s)$)	13.8 (0.8)	14.3 (1.1)	14 (0.8)	14.2 (1.2)	t = 0.-95, *p* = 0.35
**Running economy**					
EE 11 km/h (kcal/min/kg, $\overline{x}(s)$)	0.19 (0.02)	0.18 (0.02)	0.19 (0.02)	0.18 (0.02)	C: F(1, 49) = 0.80, *p* = 0.37
EE 12 km/h (kcal/min/kg, $\overline{x}(s)$)	0.20 (0.02)	0.20 (0.02)	0.20 (0.02)	0.20 (0.02)	S: F(2, 98) = 448.08, *p* < 0.001
EE 13 km/h (kcal/min/kg, $\overline{x}(s)$)	0.22 (0.02)	0.21 (0.02)	0.22 (0.02)	0.21 (0.02)	CxS: F(2, 98) = 0.78, *p* = 0.46
**Training**					
Days-a-week (n, $\overline{x}(s)$)	5 (1)	4 (2)	4 (1)	4 (2)	U = 482.5, *p* = 0.61
Weekly volume (km, $\overline{x}(s)$)	57.1 (22.6)	36.2 (23.3)	45.8 (24.7)	44 (25.8)	U = 484, *p* = 0.60
**Performance**					
10K time (mm:ss, $\overline{x}(s)$)	42:27 (05:04)	40:17 (03:56)	41:44 (04:11)	40:34 (04:57)	t = 0.99, *p* = 0.32

**Notes.**

n: count; $\overline{x}$: mean; (*s*): standard deviation.

The All column is divided in females (F) and males (M).

EE: energy expenditure.

In the cluster comparison, X^2^: Chi-squared test; U: Mann–Whitney U test for independent groups; t: *t*-test for independent groups; F: 2-way analysis of variance (ANOVA) with repeated measures for the main effects of cluster (C), and speed (S) and the interaction effect of these two (CxS). Note that S here refers to the speed during the incremental protocol in Rivadulla et al. (2024).

The experimental protocol required participants to run on a treadmill (J100; Powerjog Inc., North Charleston, SC, USA) at constant speed until volitional exhaustion. The treadmill was set at 1% gradient (Jones & Doust, 1996) and at a speed 5% faster than the lactate threshold speed computed during the incremental test carried out 7 days earlier (Rivadulla et al., 2024) *via* the *D*_*max*_ method (Cheng et al., 1992). This speed was selected to place participants in the severe intensity domain (Morton, 2006), leading them to exhaustion in a time which was representative of long distance running (Zandbergen et al., 2023) but reasonable for a camera-based motion capture laboratory study. The test started with a 5-minute warm-up in which runners were ramped up to the speed selected to complete the trial to exhaustion. After a 5-minute rest, the trial to exhaustion started. Throughout the test, participants were provided with frequent verbal encouragement to obtain their maximum effort (Andreacci et al., 2010). Additionally, participants were instructed to avoid intense physical activity, alcohol, and caffeine for 48 h prior to testing and to have their last meal 2 h before the test, to obtain participants’ maximal performance and standardise testing conditions. Participants wore their preferred running shoes to complete the test. Tests took place in the Applied Biomechanics Suite at the University of Bath, where air flow, temperature and humidity were controlled *via* mechanical ventilation (average ± standard deviation temperature = 21.7 ± 1.3 °C and relative humidity = 36.9 ± 7.6%).

### Data collection and processing

The same procedure outlined in Rivadulla et al. (2024) was used for data collection and processing. Full body kinematics were recorded at 200 Hz using a 16-camera motion capture system (Oqus, Qualisys, Sweden) and retroreflective spherical markers (see Appendix). Optical motion capture is widely regarded as the gold standard for kinematic analysis, with sub-millimetre accuracy in marker tracking. To enhance reliability, all markers were placed by the same experienced investigator and system calibration was performed prior to each testing session as per manufacturer’s guidelines. Marker trajectories were low-pass filtered (Butterworth, 4th order, zero-lag) with a cut off frequency of 10 Hz (Fukuchi, Fukuchi & Duarte, 2017). A six degrees-of-freedom modelling approach was used to estimate body segments’ position and orientation (Visual3D™; C-Motion, C-Motion, Inc, Rockville, MD, USA). To prevent the loss of data from motion capture failures due to the length of the trials collected, the tests were recorded in batches of 9 min with 1 min and 30 s gaps in between recordings to allow data saving. Aberrant strides whilst getting on and off the treadmill (first and last 10 s of the trial respectively) were rejected. Foot strike and toe off were detected using the FootNet algorithm (Rivadulla et al., 2021). Recordings were sliced in strides defined from right foot-strike to right foot-strike, and strides were time-normalised to 201 data points (average stride duration was 137 ± 7 frames, approximately 685 ms). Three time-segments were selected for each trial to exhaustion: start (0%), middle (50%) and end (100%) of run. The 50 closest available strides to each of these time landmarks were selected for analysis.

To describe running technique, both spatiotemporal (0D) and time-series (1D) variables were employed including stride frequency (calculated as 1/stride time) normalised to leg length, duty factor (the ratio of contact time to stride time), the vertical center of mass displacement normalised to leg length (vCOM), the trunk modelled as a single segment relative to the pelvis (trunk-to-pelvis), hip, knee and ankle joint angles in the sagittal plane, and the pelvis segment angle in the sagittal plane. These variables were the same we used in our previous work where we identified the two clusters of runners (Rivadulla et al., 2024). We based our selection of variables to describe running technique on the importance of sagittal plane motion for energy expenditure during running, which relates to running economy, one of the key determinants of running performance. Previous work has found that that the majority of metabolic energy during running is used for body weight support and forward propulsion (80%), and leg swing (7%) (Arellano & Kram, 2014). Movements in the frontal and transverse planes were excluded due to their higher susceptibility to errors from marker placement and soft tissue movement (Osis et al., 2016; Reinschmidt et al., 1997). Coordination variability was also calculated for the trunk-to-pelvis –hip, hip –knee and knee –ankle couplings using the angular velocity ellipse area method (Stock et al., 2025). For each coupling of a given participant and time segment, coordination variability was calculated as follows: for a given time point, the covariance matrix of all the included strides within a time segment is computed. The eigenvalues of this covariance matrix are then calculated and scaled to form an ellipse where 95% of the hypothetical new observations would be expected to fall. The area of this ellipse represents the coordination variability of a given joint coupling at that specific time point. The process is then repeated for every time point within the registered stride cycles (*i.e.,* 201).

Participants reported their perceived exertion every 10 min during the test and once more at the conclusion, using the 6–20 Borg RPE scale (Borg, 1982). Blood lactate levels were measured immediately after the test using a Lactate Plus analyser (Nova Biomedical, Waltham, MA, USA), which required approximately 0.7 µl of blood per sample. A portable metabolic system (K5; Cosmed, Rome, Italy) was employed to monitor breath-by-breath gas exchange, including oxygen uptake ($\dot {V}{O}_{2}$), respiratory quotient (RQ $=\dot {V}C{O}_{2}/\dot {V}{O}_{2}$), breathing rate, and tidal volume throughout the trial. Previous studies demonstrated the excellent reliability of this device against a metabolic simulator (Guidetti et al., 2018). Both the lactate and metabolic analysers were calibrated before each session in accordance with the manufacturers’ instructions. To help participants acclimate to the equipment, the respiratory mask was fitted five minutes prior to the warm-up. Gas exchange data were reviewed in 30-second segments, and any breaths falling outside ±2 standard deviations from the local mean were excluded as outliers.

### Data analysis

Running kinematics and coordination variability were statistically compared using nonparametric SPM and conventional two-way analysis of variance (ANOVA) with repeated measures for the 1D and 0D variables, respectively (α = 0.05). Significant main (cluster and exhaustion) and interaction effects (cluster x exhaustion) were followed up with *post hoc* Bonferroni corrected *t*-tests. Clusters were also compared for their time to exhaustion, demographics (sex and age), anthropometrics (height, body mass, trunk, leg, thigh, shank and foot length, and pelvis width), physiological capacity ($\dot {V}{O}_{2peak}$, lactate threshold and running economy at 11, 12 and 13 km/h during the incremental test as per our previous study (Rivadulla et al., 2024), training (training days per week and weekly volume) and performance (10K time). T-tests were used when the normality assumption was met according to the Shapiro–Wilk test and Mann–Whitney U tests when data were not normally distributed for every continuous and ordinal 0D variable. Categorical variables were compared using the X^2^ test. Effect sizes (Cohen’s d, (Cohen, 1988)) and 95% confidence intervals (95% CI [lower bound, upper bound]) were reported for pairwise 0D comparisons. All the analyses were performed on Python 3.8, using the pingouin 0.5.3 and spm1d 0.4.20 libraries for statistics. Data, environment file and code to replicate the current results can be found in https://github.com/adrianrivadulla/fatigue_runners.

## Results

**Figure 1:**
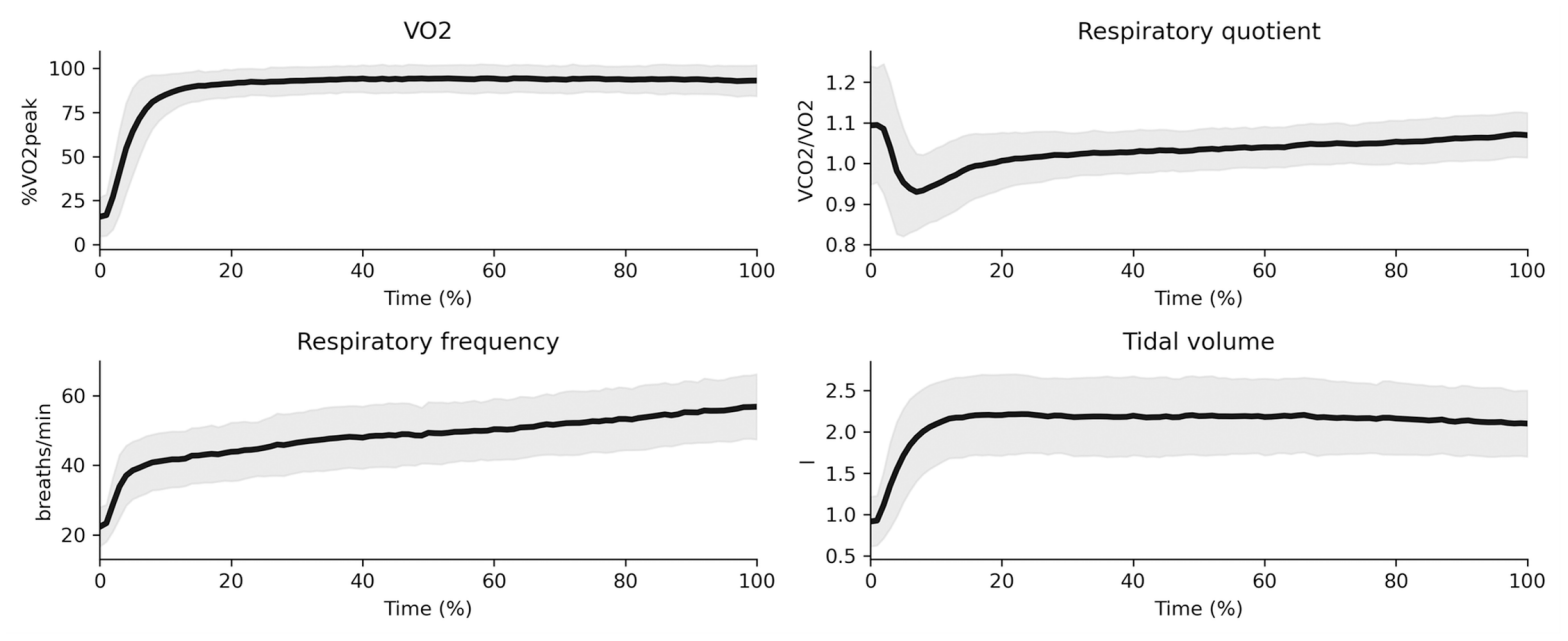
Average (and standard deviation cloud) respiratory variables throughout the trial to exhaustion (0–100%) for the entire sample.

**Figure 2:**
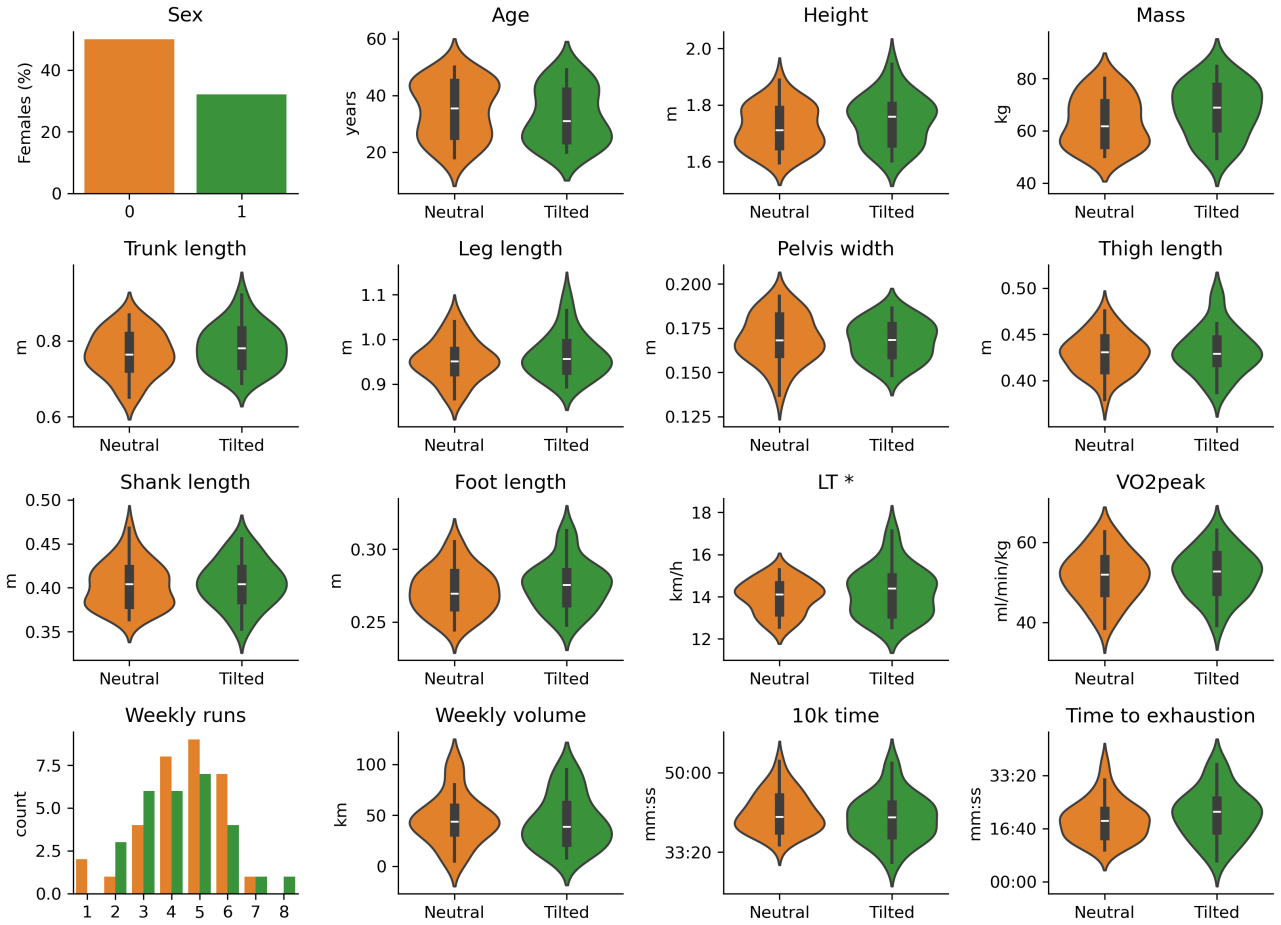
Demographics, anthropometrics, physiological capacity, training and performance variables by cluster. No significant differences were found between clusters. Full statistics are reported in Table 1.

The median RPE at the conclusion of the test was 20, with 95% of the participants reporting RPE ≥ 19. The mean lactate concentration upon termination of the trial was 7.6 ± 2 mmol/l. $\dot {V}{O}_{2}$ reached a mean of ∼95 ± 5% of the $\dot {V}{O}_{2peak}$ identified in the incremental test (Rivadulla et al., 2024), with respiratory quotient > 1 which increased throughout the trial. Respiratory frequency also increased linearly through the run whereas tidal volume remained stable (Fig. 1). The average time to exhaustion for all the participants was 20:07 ± 06:59 (min:sec), covering an average distance of 5,021 ± 1,931 m. There were no significant differences in time to exhaustion between clusters (t = −0.97, *p* = 0.33). Clusters showed no significant differences in demographics, anthropometrics, physiological capacity, training and performance (Fig. 2 and Table 1).

### Cluster main effects and cluster x exhaustion interaction effects on running kinematics

**Figure 3:**
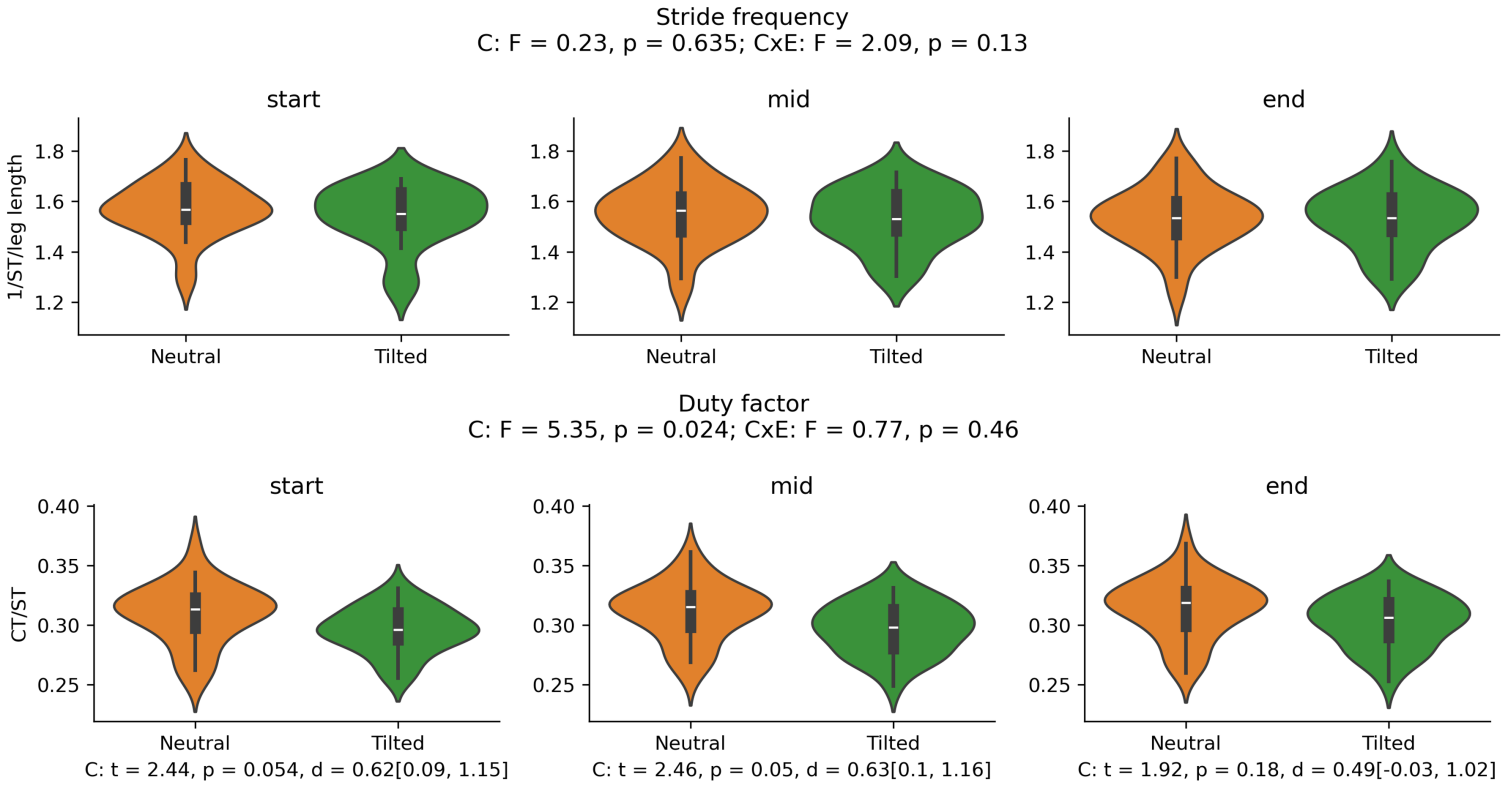
0D variables. Violin plots by cluster for stride frequency and duty factor at the start, middle and end segment of the trial respectively. Main effects for cluster (C), and cluster x exhaustion (E) interaction effect (CxE) are reported in the titles. A significant cluster effect on duty factor was found and *post hoc* tests results are reported underneath each graph. ST in the Stride Frequency y label corresponds to stride time. Leg length in the stride frequency normalisation is measured in meters. CT and ST in the Duty Factor y label correspond to contact time and stride time, respectively.

**Figure 4:**
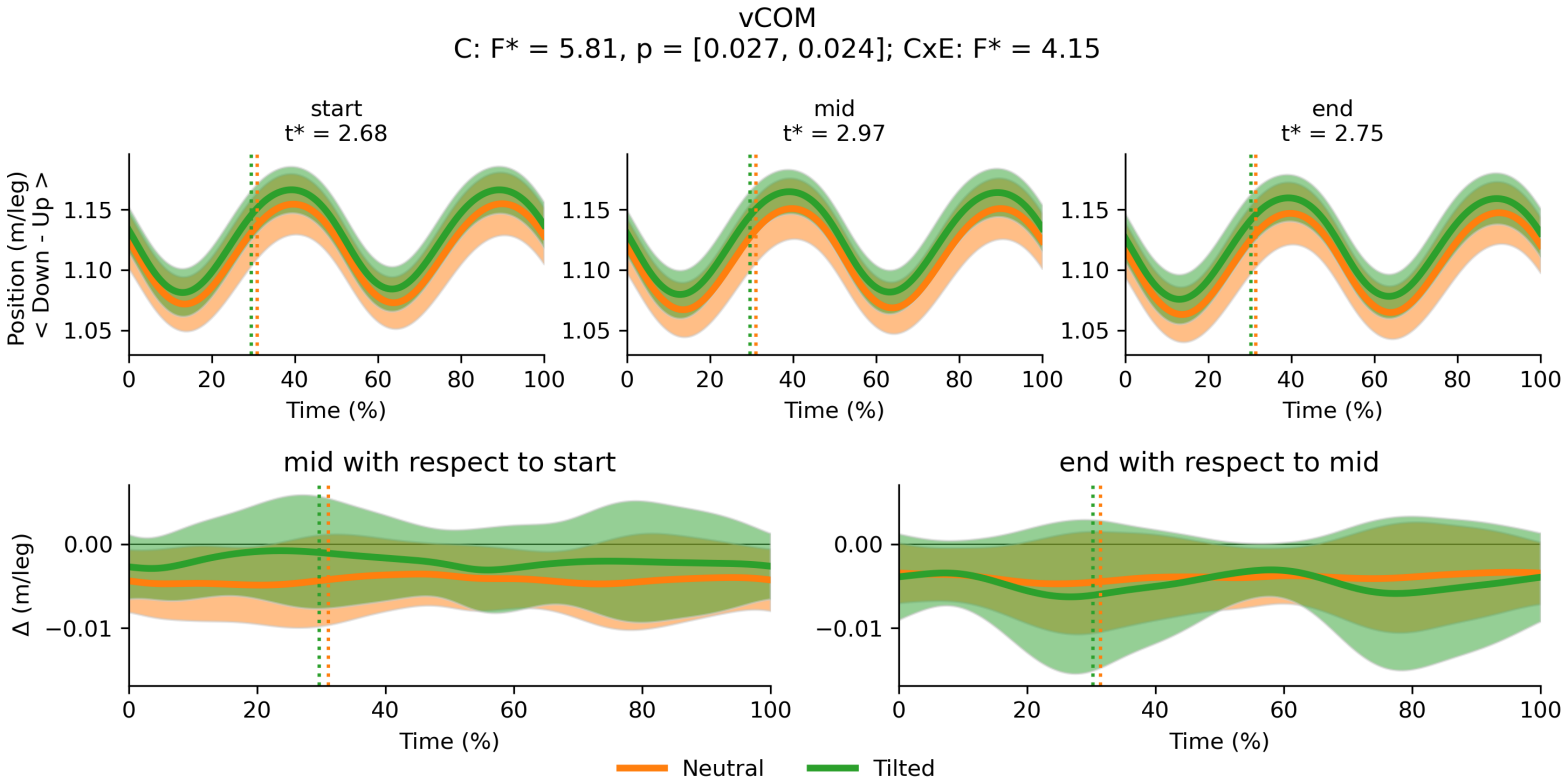
Cluster (C) and cluster x exhaustion (E) interaction effects (CxE) for the vCOM during the stride (foot-strike to foot-strike, 0–100%). Top row: average vCOM displacement (and standard deviation cloud) for each cluster. Bottom row: Average change in vCOM displacement (and standard deviation cloud) for each cluster. Vertical dotted lines indicate the end of the average contact phase of the stride.

**Figure 5:**
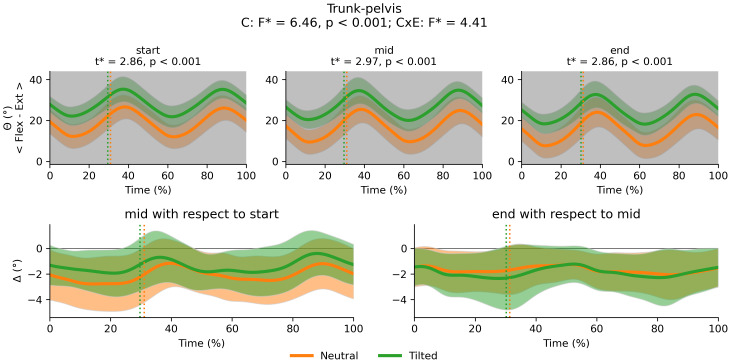
Cluster (C) and cluster x exhaustion (E) interaction effects (CxE) for the trunk-to-pelvis angle during the stride (foot-strike to foot-strike, 0–100%). Top row: Average trunk-to-pelvis angle (and standard deviation cloud) for each cluster. Areas of the gait cycle where significant differences were found in *post-hoc* analysis following a significant cluster effect are highlighted in grey. Bottom row: Average change in trunk-to-pelvis angle (and standard deviation cloud) for each cluster. Vertical dotted lines indicate the end of the average contact phase of the stride.

**Figure 6:**
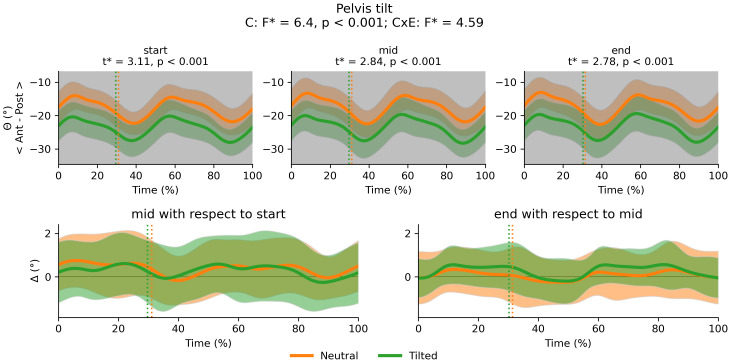
Cluster (C) and cluster x exhaustion (E) interaction effects (CxE) for the pelvis tilt angle during the stride (foot-strike to foot-strike, 0–100%). Top row: Average pelvis angle (and standard deviation cloud) for each cluster. Areas of the gait cycle where significant differences were found in *post hoc* analysis following a significant cluster effect are highlighted in grey. Bottom row: average change in pelvis angle (and standard deviation cloud) for each cluster. Vertical dotted lines indicate the end of the average contact phase of the stride.

**Figure 7:**
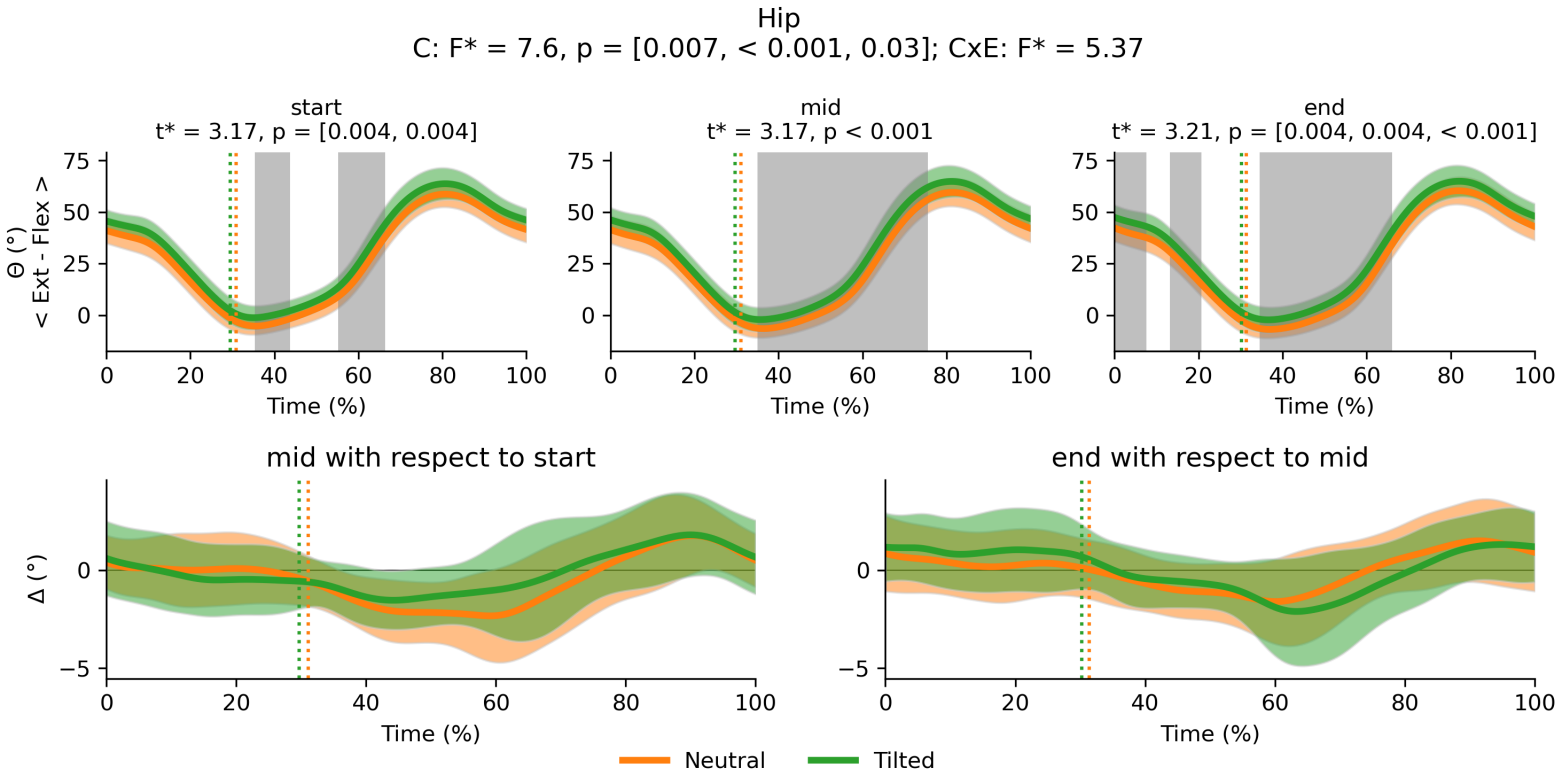
Cluster (C) and cluster x exhaustion (E) interaction effects (CxE) for the hip angle during the stride (foot-strike to foot-strike, 0–100%). Top row: Average hip angle (and standard deviation) cloud for each cluster. Areas of the gait cycle where significant differences were found in *post hoc* analysis following a significant cluster effect are highlighted in grey. Bottom row: Average change in hip angle (and standard deviation cloud) for each cluster. Vertical dotted lines indicate the end of the average contact phase of the stride.

**Figure 8:**
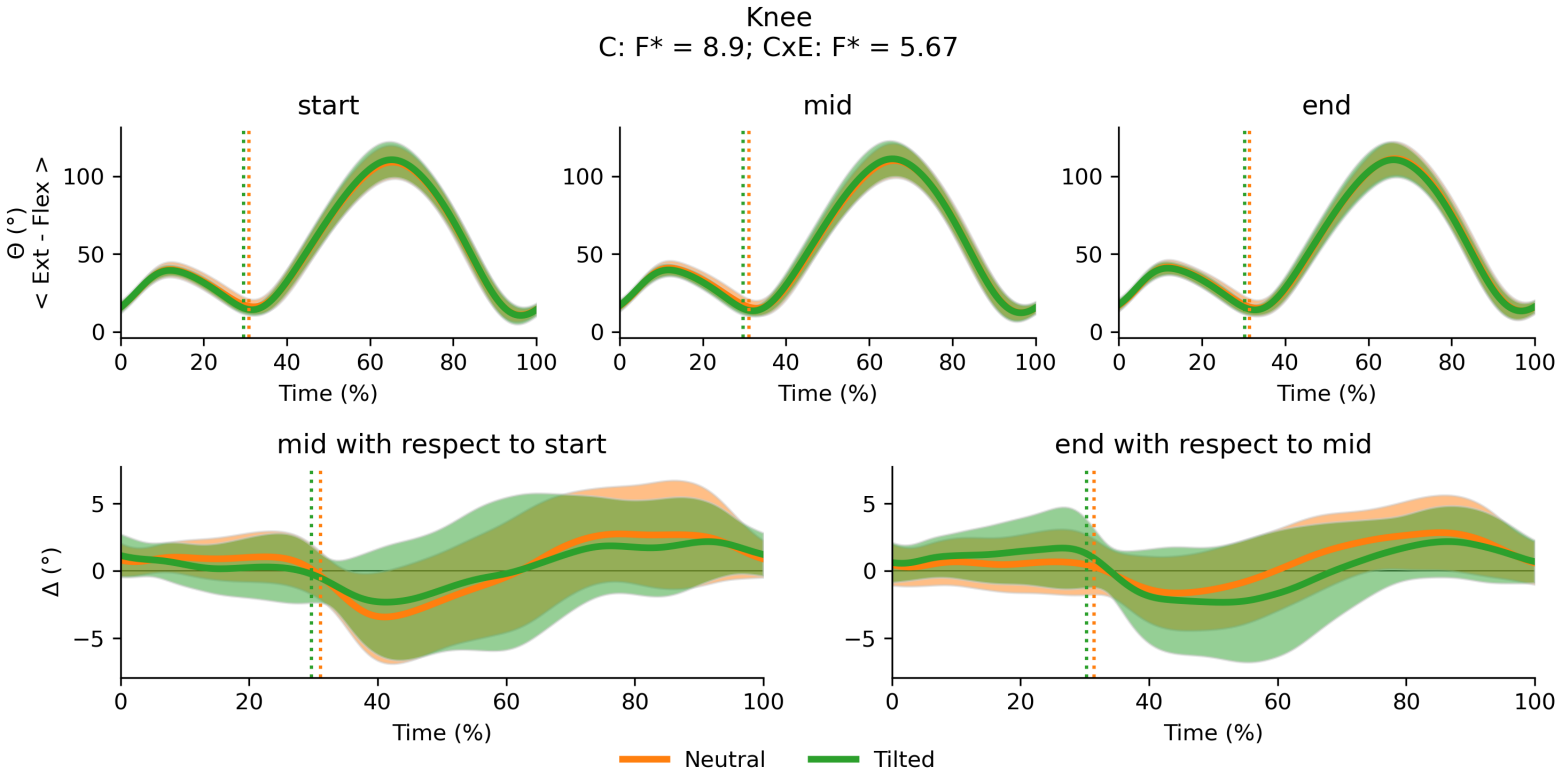
Cluster (C) and cluster x exhaustion (E) interaction effects (CxE) for the knee angle during the stride (foot-strike to foot-strike, 0–100%). Top row: Average knee angle (and standard deviation cloud) for each cluster. Bottom row: Average change in knee angle (and standard deviation cloud) for each cluster. Vertical dotted lines indicate the end of the average contact phase of the stride.

**Figure 9:**
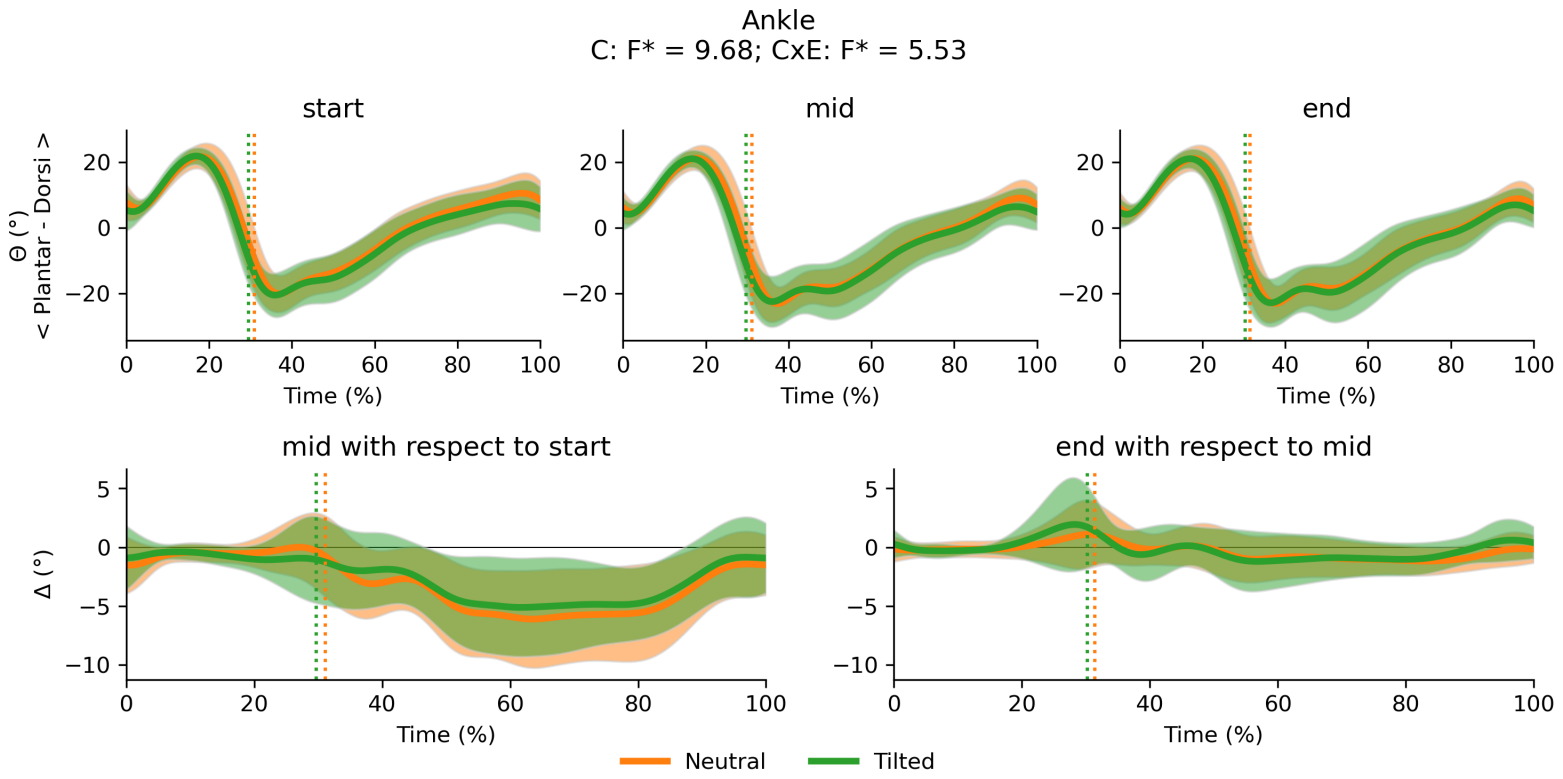
Cluster (C) and cluster x exhaustion (E) interaction effects (CxE) for the ankle angle during the stride (foot-strike to foot-strike, 0–100%). Top row: Average ankle angle (and standard deviation cloud) for each cluster. Bottom row: Average change in ankle angle (and standard deviation cloud) for each cluster. Vertical dotted lines indicate the end of the average contact phase of the stride.

There was a main effect of cluster on duty factor (Fig. 3), vCOM (Fig. 4), trunk-to-pelvis (Fig. 5), pelvis (Fig. 6) and hip angle (Fig. 7). The tilted pelvis cluster exhibited smaller duty factor (Fig. 3) less trunk-to-pelvis flexion (Fig. 5) and more pelvic tilt (Fig. 6) throughout the stride and at every segment of the trial. The tilted pelvis cluster also showed greater hip flexion mainly during the swing phase during every segment of the trial and at several phases during the stance at the end of the trial too (Fig. 7). Differences in vCOM could not be confirmed in *post hoc* analyses (Fig. 4). There were no significant cluster effects for the knee (Fig. 8) and ankle angles (Fig. 9). There were no significant interactions between cluster and exhaustion. Full results are reported in the corresponding figures.

### Exhaustion main effects on running kinematics

**Figure 10:**
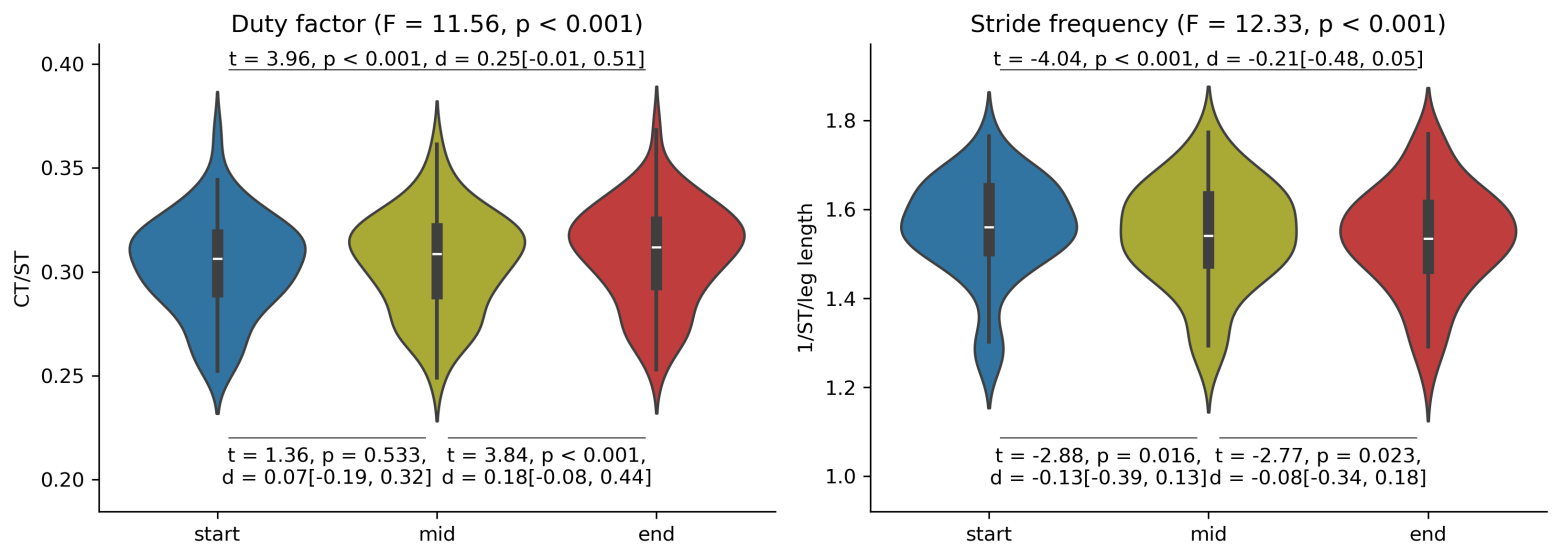
Main effect of exhaustion on duty factor (CT: contact time; ST: stride time), and stride frequency. Violin plots for the entire sample for stride frequency and duty factor at the start, middle and end segment of the trial respectively. Significant effects of exhaustion on duty factor and stride frequency were found and *post hoc* tests results for each comparison are reported.

**Figure 11:**
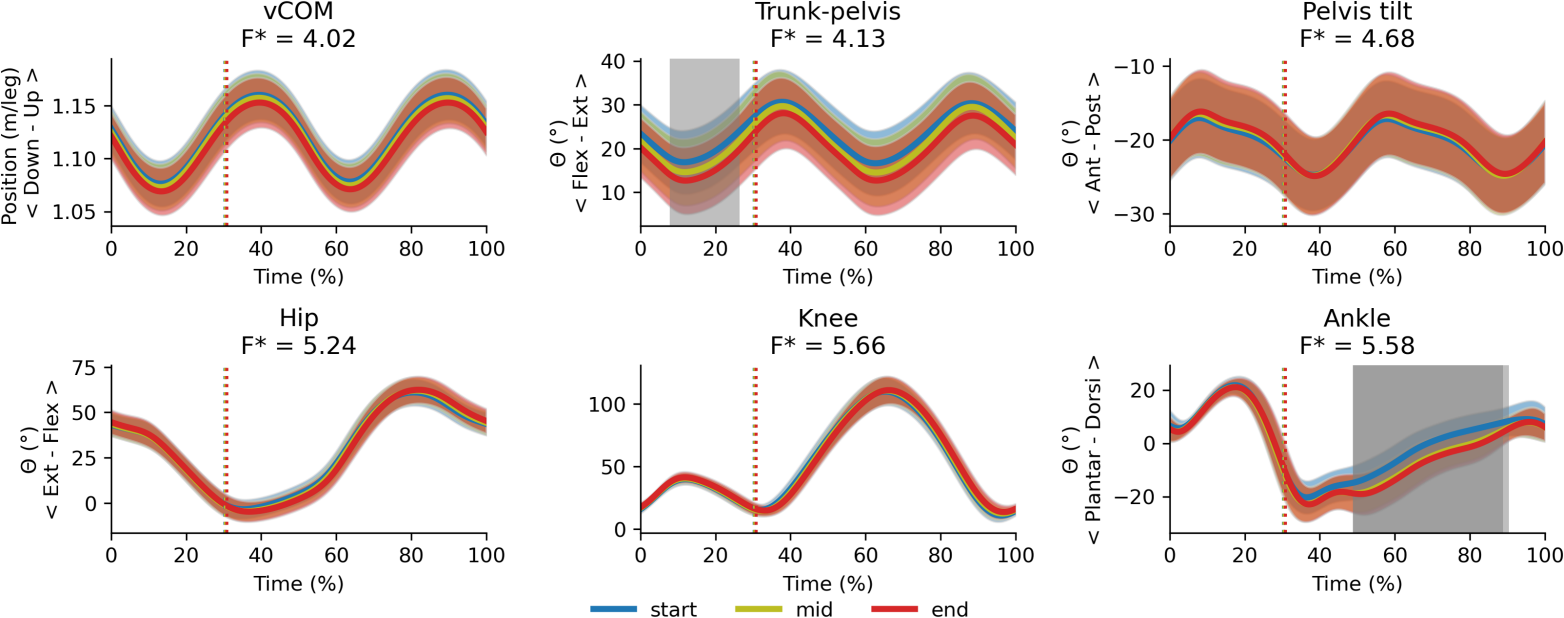
Main effect of exhaustion on 1D variables. Average (and standard deviation cloud) stride pattern (0–100% of time) for the entire sample at the start, middle and end segment of the trial respectively. Vertical dotted lines represent the average toe-off for each trial segment. Following significant main effects of exhaustion, *post hoc* tests revealed greater trunk-to-pelvis flexion at end of the trial (8–26% of the stride, Δ =  − 4°, t* = 2.96, *p* = 0.005) compared with the start and greater plantar flexion at the middle (49–89% of the stride, Δ =  − 5°, t* = 3.16, *p* < 0.001) and end of the trial (49–91% of the stride, Δ =  − 6°, t* = 3.17, *p* < 0.001) compared with the start as indicated by the grey patches.

There were significant main effects of exhaustion on stride frequency, duty factor (Fig. 10), trunk-to-pelvis and ankle angle (Fig. 11). *Post hoc* analysis revealed that all participants increased their duty factor at the end of the trial compared with the start and the middle of the trial and decreased their stride frequency significantly between each segment of the trial (Fig. 10). Additionally, participants increased their trunk-to-pelvis flexion during the central part of the stance phase at the end of the trial with respect to the start, as well as plantar flexion during the swing phase at the middle and end of the trial with respect to the start (Fig. 11).

### Cluster and exhaustion main and interaction effects on coordination variability

**Figure 12:**
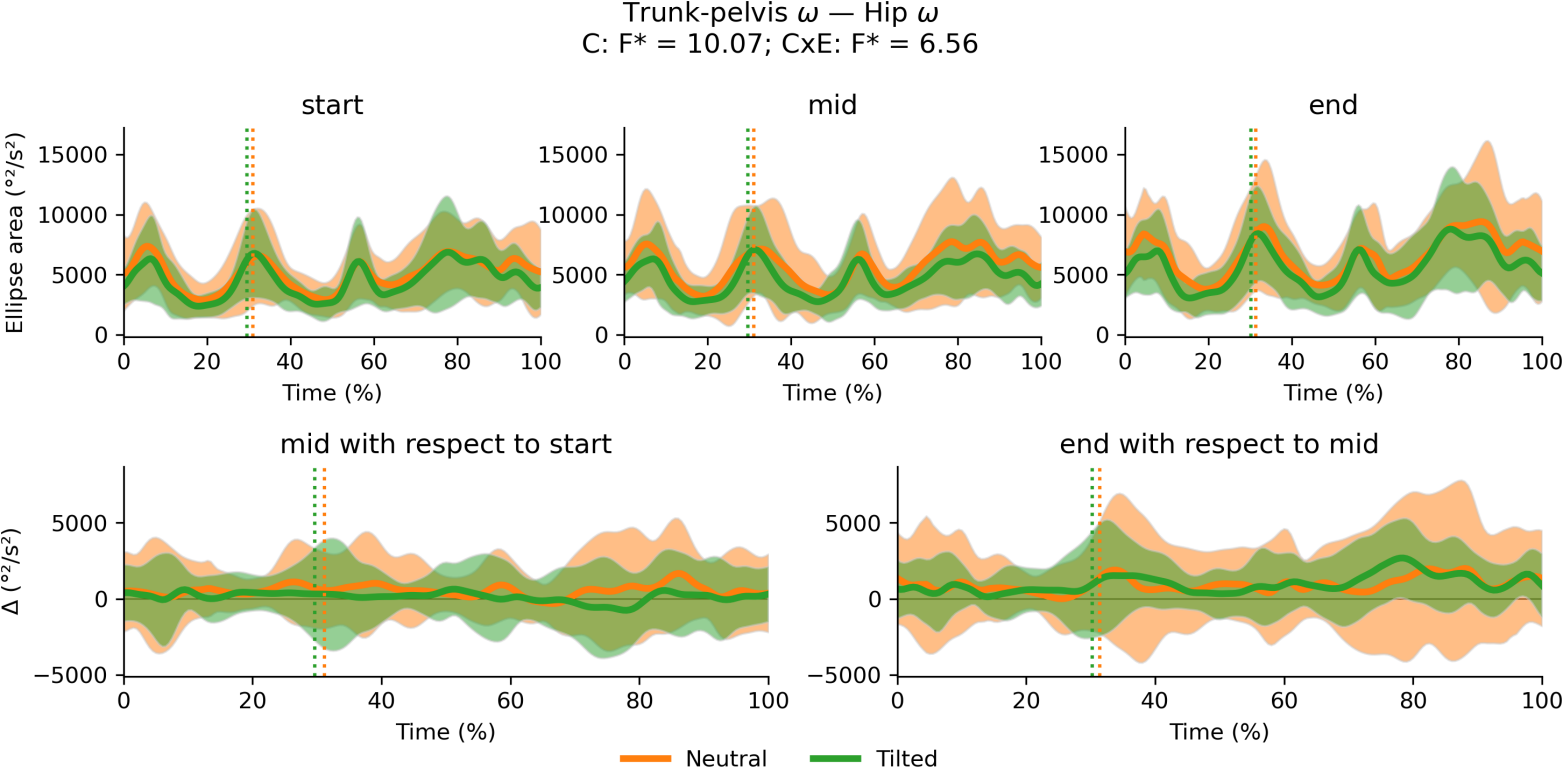
Cluster (C) and cluster x exhaustion (E) interaction effects (CxE) for the trunk-to-pelvis x hip joint coupling coordination variability during the stride (foot-strike to foot-strike, 0–100%). Top row: Average ellipse area (and standard deviation cloud) for each cluster. Bottom row: Average change in ellipse area (and standard deviation cloud) for each cluster. Vertical dotted lines indicate the end of the average contact phase of the stride.

**Figure 13:**
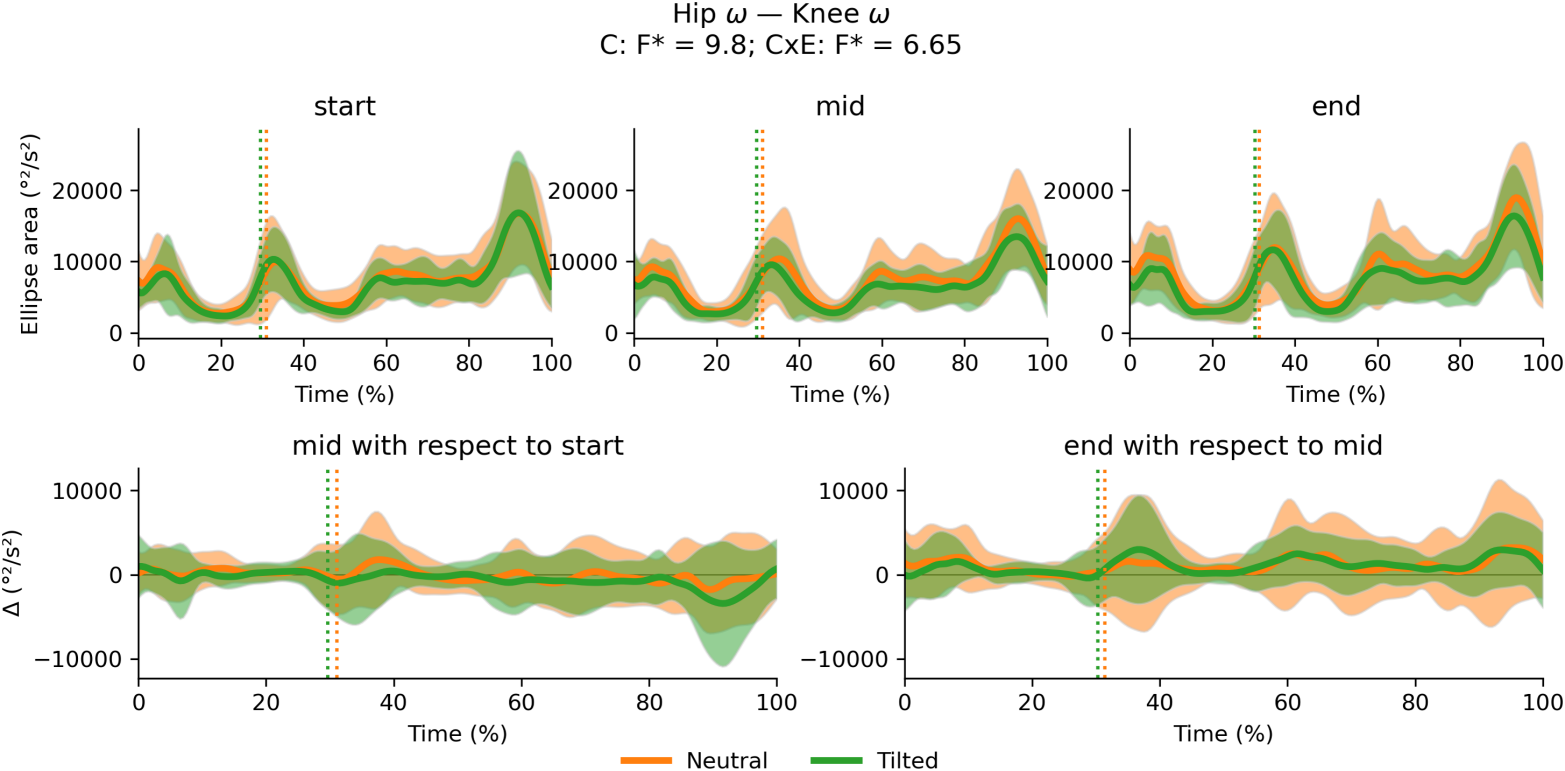
Cluster (C) and cluster x exhaustion (E) interaction effects (CxE) for the hip –knee joint coupling coordination variability during the stride (foot-strike to foot-strike, 0–100%). Top row: Average ellipse area (and standard deviation cloud) for each cluster. Bottom row: Average change in ellipse area (and standard deviation cloud) for each cluster. Vertical dotted lines indicate the end of the average contact phase of the stride.

**Figure 14:**
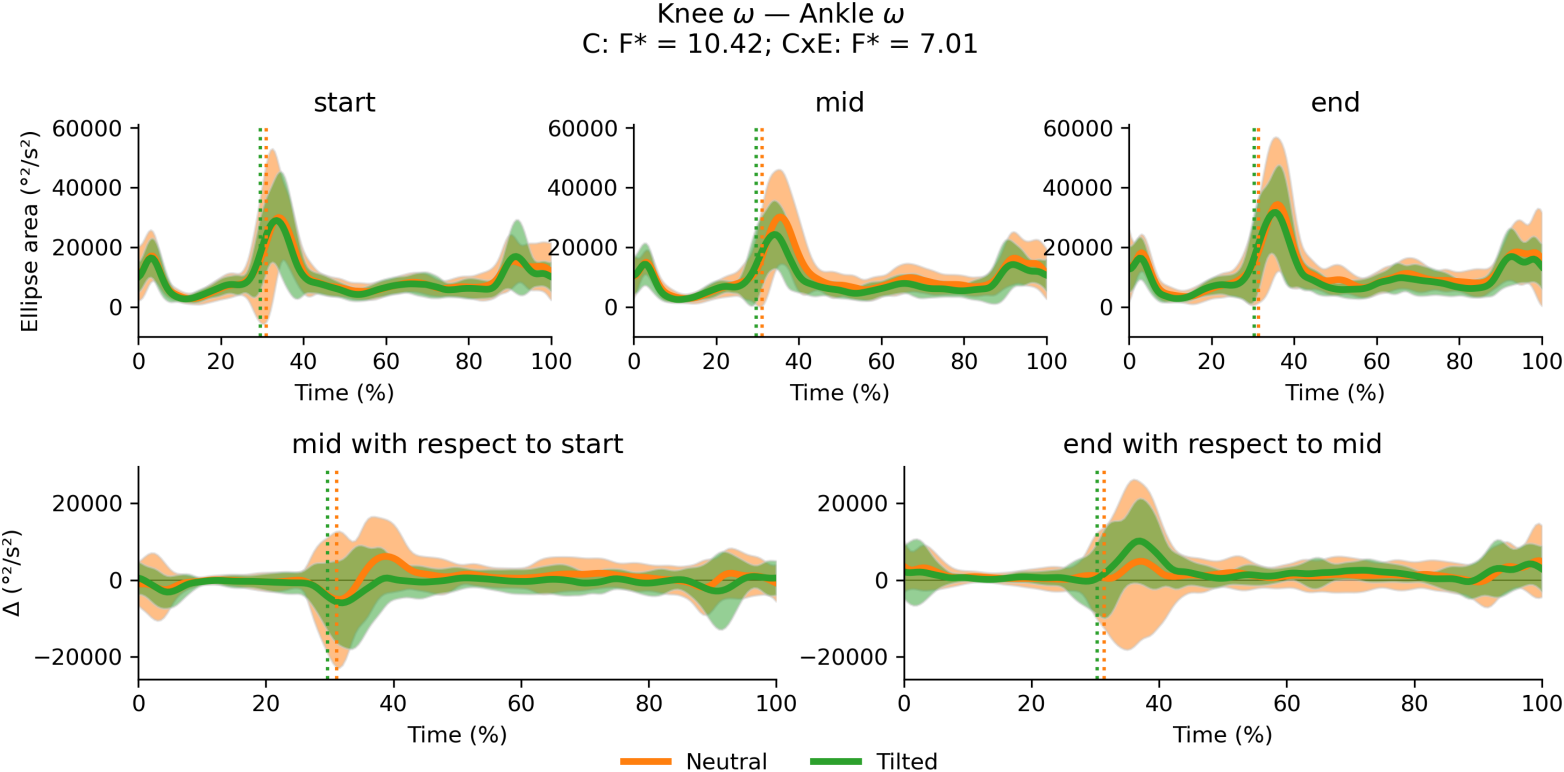
Cluster (C) and cluster x exhaustion (E) interaction effects (CxE) for the knee–ankle joint coupling coordination variability during the stride (foot-strike to foot-strike, 0–100%). Top row: Average ellipse area (and standard deviation cloud) for each cluster. Bottom row: Average change in ellipse area (and standard deviation cloud) for each cluster. Vertical dotted lines indicate the end of the average contact phase of the stride.

**Figure 15:**
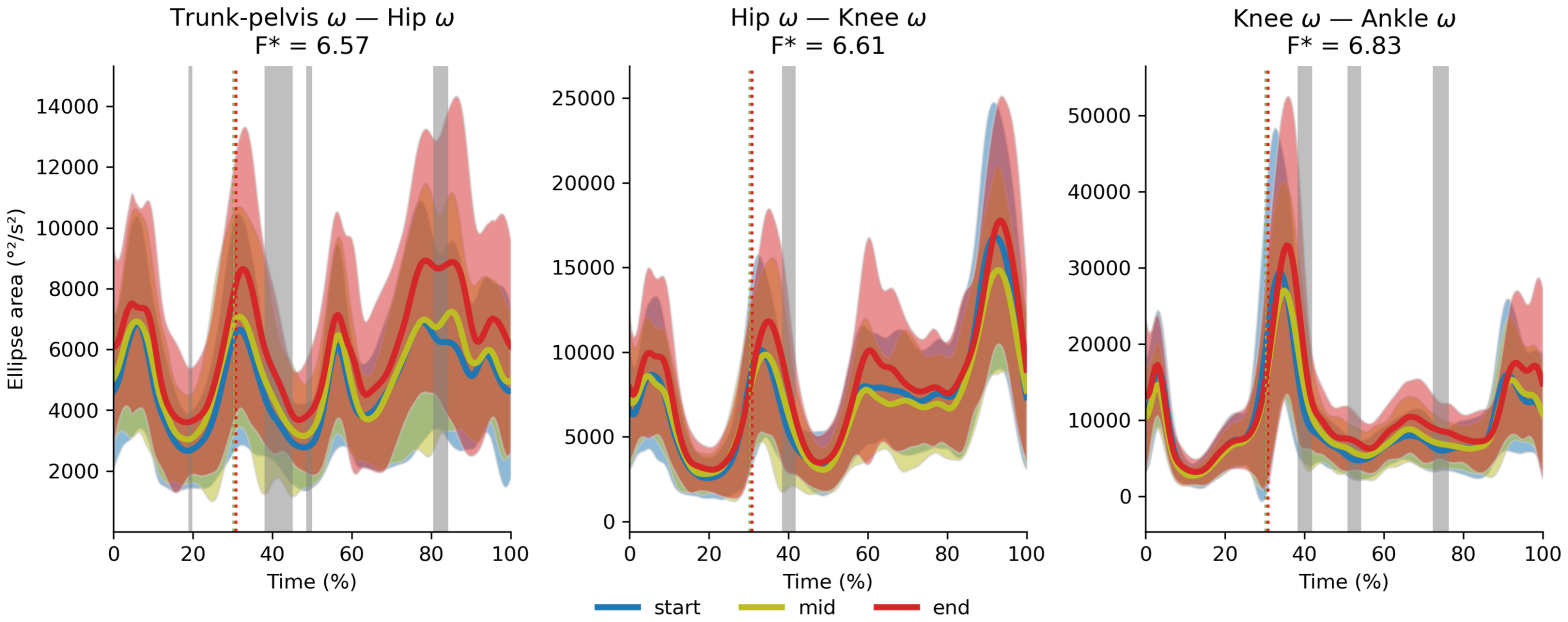
Main effect of exhaustion on coordination variability. Average (and standard deviation cloud) stride pattern (0–100% of time) for the entire sample at the start, middle and end segment of the trial respectively. Vertical dotted lines represent the average toe-off for each trial segment. Following significant main effects of exhaustion, *post hoc* tests revealed greater coordination variability when comparing the end segment with the start segment for the trunk-to-pelvis–hip (t* = 3.5, *p* < 0.001, 39–44% of the stride; *p* = 0.005 81–83% of the stride), the hip–knee (t* = 3.57, *p* = 0.005, 39–41% of the stride) and the knee –ankle (t* = 3.51, *p* = 0.001, 38–42% of the stride; *p* = 0.001, 51–54% of the stride; *p* < 0.001, 72–76% of the stride) couplings as indicated by the grey patches.

There were no significant cluster effects or interactions between cluster and exhaustion for the trunk-to-pelvis–hip (Fig. 12), hip–knee (Fig. 13) and knee–ankle (Fig. 14) joint couplings coordination variability. There were significant main effects of exhaustion on the coordination variability of the trunk-to-pelvis–hip, hip–knee and knee–ankle joint couplings (Fig. 15). *Post hoc* analysis revealed that all participants increased coordination variability in the trunk-to-pelvis–hip coupling during the early and late swing phase, in the hip–knee coupling during the early swing phase, and in the knee –ankle coupling in several sections of the swing phase, when comparing the end with the start time segment.

## Discussion

The aim of this study was to compare the changes in running technique during a treadmill run to exhaustion amongst runners who had been grouped into the neutral and tilted pelvis clusters based on their preferred technique (Rivadulla et al., 2024). We contributed to overcoming the limitations identified in previous studies by conducting a comprehensive biomechanical assessment of running which includes spatiotemporal, kinematic and joint coordination variability time series analysis on an extensive sample of runners; and controlling for the intensity of the run on an individual basis. The two clusters showed no significant differences in their time to exhaustion and maintained their between-cluster kinematic differences throughout the trial, with the tilted pelvis cluster showing smaller duty factor, more trunk-to-pelvis extension, anterior pelvic tilt and hip flexion compared with the neutral pelvis cluster. On average, all participants reduced stride frequency, and increased duty factor, trunk-to-pelvis flexion during the stance, plantar flexion and coordination variability in the trunk-to-pelvis–hip, hip–knee and knee–ankle couplings during the swing phase as the trial to exhaustion went on. However, these changes were small suggesting that only minor adjustments to technique occur when exhaustion is reached in ∼20 min. Despite the differences in their preferred technique, runners from different clusters did not show evidence of differential changes to their technique during the trial, rejecting our initial hypothesis.

We assessed the changes in running technique during a treadmill run to exhaustion at a speed 5% faster than the individual-specific lactate threshold, similar to Möhler, Fadillioglu & Stein (2021). The individual calculation of lactate threshold was used to match exercise intensity since this can affect the changes to running technique (Apte et al., 2021). Exhaustion times occurred in ∼20 min, with runners covering ∼5 km and reporting perceptual and physiological symptoms of fatigue. Participants rated their effort as extremely hard or maximal exertion at the end of the trial and lactate concentration levels in blood reached 7.6 ± 2 mmol/l. These lactate values were on average greater than those observed in maximal lactate steady state running studies (Fontana, Boutellier & Knöpfli-Lenzin, 2009). Participants also reached a $\dot {V}{O}_{2}$ ≥ 90% of the $\dot {V}{O}_{2peak}$ identified during the maximal incremental test used to determine their lactate threshold (Rivadulla et al., 2024), similar to that measured during 5K races (Ramsbottom, Nute & Williams, 1987). The respiratory quotient kept increasing throughout the trial for all participants as a result of anaerobic metabolism (Spriet, 2022). We also observed participants increased ventilation *via* an initial rapid increase of tidal volume that then plateaued or even reduced, and an initial rapid rise in respiratory frequency, which kept increasing linearly to the end of the trial. These symptoms are typical of the severe intensity domain (Morton, 2006) and clearly indicate runners’ considerable effort (Nicolò, Marcora & Sacchetti, 2016).

This study included a subset of the entire sample where the neutral and tilted pelvis clusters were identified (Rivadulla et al., 2024). The participants from both groups included in this study remained homogeneous for their anthropometrics, physiological capacity, training and performance variables. They showed no differences in their time to exhaustion and maintained their baseline differences in their preferred technique (Rivadulla et al., 2024) regarding pelvic tilt, trunk-to-pelvis and hip angle, and duty factor at the start of the trial to exhaustion. Preserving their distinct running technique is consistent with runners’ theoretical reluctancy to depart from their preferred movement path (Nigg, 2001). Whilst runners exhibited changes in their technique throughout the trial, these did not appear to be influenced by the cluster they belonged to, rejecting our hypothesis. As the trial progressed, participants from both clusters reduced their stride frequency on average, which required a reciprocal increase in stride length to maintain running speed. This seems congruent with the trends for a lower cadence (steps/min), longer contact and flight time identified in a review of similar treadmill testing conditions (Apte et al., 2021). It is worth noting that lower stride frequency has been associated with worse running economy, which is a key determinant of running performance (van Hooren et al., 2024). Participants also increased duty factor thus, contact times got relatively longer with respect to swing time within the stride similar to Möhler, Fadillioglu & Stein (2021). Runners also increased trunk flexion with respect to the pelvis during the stance throughout the trial (∼4° on average) and plantar flexion during the swing phase (∼6° on average), similar to the changes reported by Maas et al. (2018). Since the pelvic tilt angle did not change significantly, we can assume that this relative trunk-to-pelvis angle translated into an increased absolute trunk forward lean angle, which is one of the most commonly observed fatigue-induced changes to running technique both for treadmill (Apte et al., 2021) and overground running (Winter, Gordon & Watt, 2017). Similar to previous studies (Bailey et al., 2020; Tazji et al., 2023), coordination variability also seemed to increase in specific sections of the swing phase as exhaustion was approached.

The observed changes in running technique may be explained by a reduction in muscle capacity and an attenuation of the forces experienced whilst running as indicated in previous literature (Apte et al., 2021). Longer strides resulting from lower stride frequency have been shown to promote shock attenuation (Mercer et al., 2003) and longer contacts also allow for greater distribution of vertical impulse and reducing peak impact forces, which has been associated with injury (Johnson et al., 2020). The decrease in peak vertical forces is usually associated with a decrease in vertical stiffness, *via* greater hip and knee flexion angles and can lead to an increase in vCOM displacement (Apte et al., 2021; Zandbergen et al., 2023). We did not find significant alterations to vCOM, hip and knee angles. It is worth noting that some of the observed adaptations may be more apparent in novice populations (Maas et al., 2018; Zandbergen et al., 2023) whereas our sample included trained/highly trained runners (McKay et al., 2022). Zandbergen et al. (2023) in their review found that novices tend to increase vCOM displacement whereas experienced runners may not, which aligns with the lack of the changes in vCOM observed in the present study. The increasing coordination variability during the test may also support the hypothesis of redistributing the load and alleviating the strain experienced by specific tissues (Hamill et al., 2006). However, differences in variability were concentrated in the swing phase. During the swing phase, the body is either unloaded during flight, or load is primarily supported by the opposite leg. Runners may therefore be prioritising control of the supporting leg at the expense of some consistency in the swinging leg. Further investigation is needed to better understand the mechanisms behind this increase in coordination variability.

Overall, runners exhibited subtle adjustments to their running technique in the present experiment. Indeed, the changes to stride frequency and duty factor from the start to the end of the trial can be described as small (d = −0.21 95% CI [−0.48–0.05] and *d* = 0.25 95% CI [−0.01–0.51], respectively) with 95% confidence intervals spanning close to 0. This suggests our test where exhaustion was reached in ∼20 min may induce minimal adjustments to technique before volitional exhaustion, similar to Maas et al. (2018). Larger changes to running technique may be more likely to occur in longer runs *e.g.*, marathon (Chan-Roper et al., 2012; Reenalda et al., 2016). From a methodological perspective it is worth noting that, there were no differences in duty factor between the start and middle of the trial, which illustrates the impact the experimental protocol might have on the final conclusions of the study: Stopping trials before reaching volitional exhaustion can overlook biomechanical changes occurring as fatigue develops (Zandbergen et al., 2023), supporting our design choice. Overall, our findings can help researchers, coaches and running technology developers identify the variables which are relevant for technique monitoring and early detection of fatigue. For example, stride frequency and duty factor have been previously proposed as global descriptors of running technique (van Oeveren et al., 2021), are sensitive to fatigue as seen in our study and previous consistent trends in the literature (Apte et al., 2021; Zandbergen et al., 2023) and are relatively easy to measure *via* affordable, non-invasive wearable technologies (van Hooren et al., 2020).

Although no cluster specific differential technique changes were observed as the test progressed, it is worth noting that some of the observed modifications throughout the trial (*e.g.*, increased duty factor, increased trunk flexion) directly impact the variables that defined the clusters. It is unclear whether these changes pose the same burden for runners in each cluster. For instance, runners in the neutral pelvis cluster naturally exhibit a more flexed trunk with respect to the pelvis. The increase in trunk flexion through the trial may be associated with fatigue of the core and postural muscles, which could place greater strain on the back muscles in this cluster compared with the tilted pelvis cluster, potentially leading to the development of back pain (Kobsar & Ferber, 2018; Warrener, Tamai & Lieberman, 2021). Further research is needed to elucidate whether increased trunk flexion with fatigue has different implications for runners in the neutral cluster compared with the tilted pelvis cluster. Similarly, exhaustion was reached in ∼20 min and the present findings would suggest the technique changes within that time appear small and not systematically different between clusters. However, this test may be a limited representation of longer distances *e.g.*, half marathon, marathon. Whether cluster specific changes would be observed over a longer run remains to be answered. This appears critical to further explore the implications of the identified clusters for running training and future research is warranted.

This study builds upon our previous work (Rivadulla et al., 2024), which involved the same participant cohort and identified the clusters used here. Consequently, the present study relies on this convenience sample. As both studies were conceptualised together and given the exploratory nature of clustering methods, it was not feasible to conduct an a priori power analysis for the current study at the time of its design. Additionally, there is a potential selection bias in that runners in close proximity to the University who were engaged with local clubs or active in social media were more likely to join the study. Due to reasons beyond our control (*e.g.*, unavailability to complete the second session within 5–10 days after the first, injury, early termination of the trial to exhaustion due to pain or discomfort), only 60 out of the 84 runners in which the original clusters were identified completed the exhaustion protocol. Nevertheless, the number of participants within each cluster and the overall sample for the exhaustion effects are larger than the samples used in the majority of previous studies who had <20 participants (Apte et al., 2021). Despite the popularity of treadmill tests for the study of running kinematic changes associated with fatigue (Apte et al., 2021), treadmills impose a fixed speed, preventing runners from slowing down and reducing stride frequency, one of the main responses to fatigue during running (Winter, Gordon & Watt, 2017). Indeed, pacing strategies to delay and minimise fatigue are deemed a key factor in running performance optimisation (Pryor et al., 2020). Caution is advised when extrapolating the current findings to overground running (Zandbergen et al., 2023). We opted for a treadmill to facilitate the use of camera-based motion capture and to compare the response to fatigue by the clusters we had identified in our previous study (Rivadulla et al., 2024), also performed on the same treadmill. The current participants had varying familiarity with testing to exhaustion on a treadmill which might help explain the variability in the time to complete the trial. With the latest developments in sensor technology and as algorithms to estimate body kinematics become more accurate, ambulatory testing in real world running conditions should be pursued to increase the validity of biomechanical running studies. These technologies are usually more accessible to runners, facilitating longitudinal study designs and greater detail to inform long term training interventions. We have also limited our analysis to stride frequency, duty factor and trunk and lower limb kinematics in the sagittal plane since these variables have been previously suggested as optimal descriptors of running technique (Van Oeveren et al., 2021) and to be consistent with the variables used to identify the running clusters (Rivadulla et al., 2024). Variables in secondary planes may also be affected by fatigue (Willwacher, Sanno & Brüggemann, 2020) but measuring such variables reliably is challenging with conventional skin-mounted marker based motion capture technologies. Additionally, the inclusion of kinetics could further elucidate biomechanical changes related to fatigue during running.

## Conclusions

Runners from the tilted pelvis cluster maintained a more extended trunk relative to the pelvis, a more anteriorly tilted pelvis, and greater hip flexion throughout the stride, and smaller duty factor during a run to exhaustion compared with the neutral pelvis cluster. Despite these differences in their preferred technique, there were no significant differences in how runners from both clusters adjusted their technique through the trial. On average, all runners decreased their stride frequency, increased their duty factor, trunk flexion during the stance phase and plantar flexion during the swing phase as the trial went on. Since the two clusters identified exhibited differences in their preferred duty factor and trunk-to-pelvis angle, the observed increase to duty factor and trunk flexion may have different biomechanical implications for each cluster which warrants further research and consideration by coaches.

## Supplemental Information

10.7717/peerj.20673/supp-1 Supplemental Information 1 Appendix

## Additional Information and Declarations

### Data Availability

The following information was supplied regarding data availability: The data and code are available at University of Bath: https://doi.org/10.15125/BATH-01550. The code is available at GitHub and Zenodo: https://github.com/adrianrivadulla/fatigue_runners. Adrian R. Rivadulla. (2025). adrianrivadulla/fatigue_runners: v1.0.1 (v1.0.1). Zenodo. https://doi.org/10.5281/zenodo.17943965.
