# Supplementary material for: Does preferred technique influence how kinematics change during a run to exhaustion?—A cluster based approach
Source: PeerJ. 2026 Feb 11;14:e20673. doi: 10.7717/peerj.20673 (PMC12906263; doi:10.7717/peerj.20673)
Supplement: Supplemental Information 1 [file peerj-14-20673-s001.docx]

**APPENDIX**

*Marker set*

| *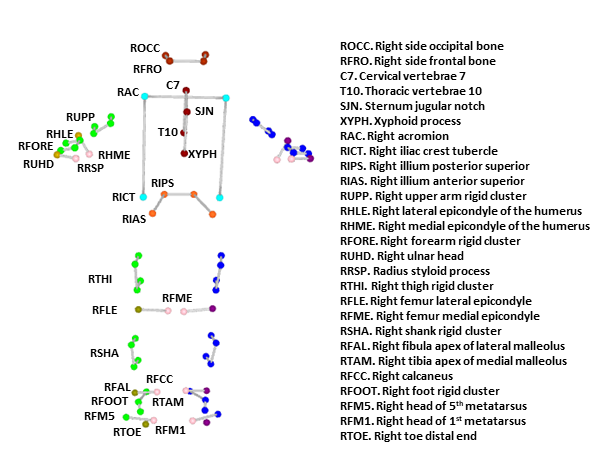* |
| --- |
| **Figure 1. Marker set used to track runners’ full body kinematics. For every right marker, there was a symmetrical marker on the left side of the body, which is shown in the stick figure, but not listed for clarity. Head markers were mounted on a headband. The rigid clusters mounted on the upper arm, forearm, thigh and shank contained four markers each. The rigid cluster mounted on the shoe tongue contained two markers. Markers and clusters were attached directly onto the skin (other than the ones mounted on the headband and shoes) using adhesive spray and double-sided tape. Clusters mounted on the limbs were additionally secured with elastic straps. The marker set included 76 markers for tracking and calibration as shown in the picture. For the running trials every marker around the lower and upper limbs joints was removed as well as the RFM1 markers.** |
